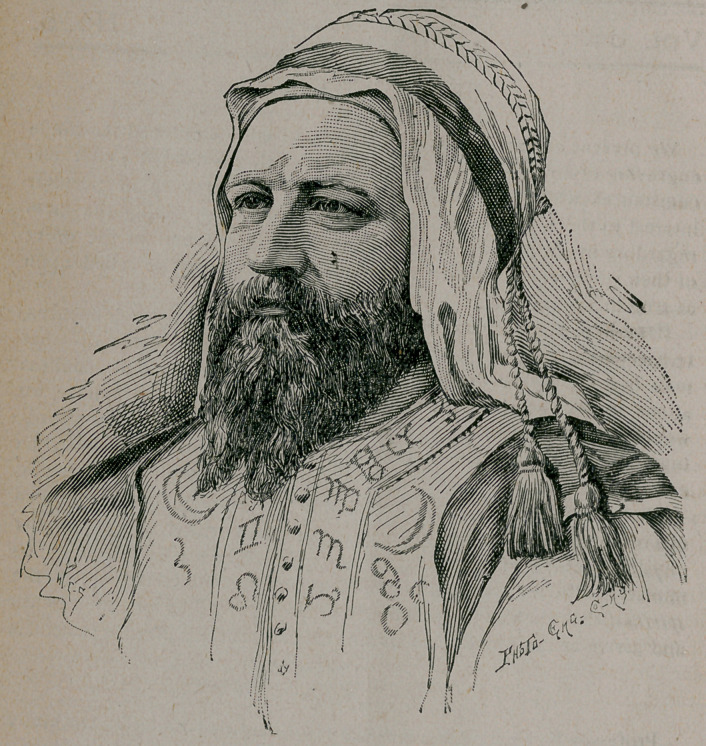# The Occult Forces

**Published:** 1887-05

**Authors:** 


### HALL'S JOURNAL OF HEALTH

TRUTH DEMANDS NO SACRIFICE; ERROR CAN MAKE NONE.

VOL. 34. MAY, 1887. No. 5.

### THE OCCULT FORCES.

### No. 2.

We present our readers as a May offering with a reduced fac-simile engraving of an original life size crayon drawing, executed under circumstances which cannot fail to render their narration of peculiar interest to them, and we shall content ourself with giving the facts regarding its production precisely as they occurred and in the order of their occurrence, with the assurance that they may be relied upon as true in every detail.

If we have any patrons who have been led by whatever influences, to accept the cold despairing doctrine that this world ends all—that man has no higher destiny than that which appertains to soulless atoms, in their everlasting round of chemical diversity and affinity, we would point them to the one truth which we present to-day, as affording indubitable evidence of the continuation of individual, conscious existance, beyond the change alike common to all, which we call death.

We have before this, taken occasion to remark upon the importance of a recognition on the part of physicians, of the spiritual, no less than the physical nature of those to whose needs they are called upon to minister. Indeed, it is oftentimes a correct understanding of the *spiritual* that enables the practioner to intelligibly diagnose the disease and arrive at the proper remedy, for oftentimes the suffering patient is

> "Not so sick" * * *
> "As she is troubled with thick—coming fancies—
> That keep her from her rest."

Professor E. D. Babbitt, in his late work entitled Religion, truthfully says: "In this age of scientific attainment, the thoughtful and cultured minds demand the demonstration of all beliefs and theories by actual facts of the living present, rather than by the traditions and old historical narrations of the past. Under their lead the world is tending inevitably to one or the other of two great divisions, first, to *materialism*, which being accustomed to look upon the coarser side of nature, and putting stress mainly upon the tangible and the visible, denies the existance of an immortal spirit in man, and hence tends to doubt the being of an infinite Father Spirit; or secondly, to Spiritualism, which being intuitional has naturally a quick perception of the finer laws of being, and building upon a large array of phenomena both objective and subjective, is led to a knowledge of spirits who have once been human beings, and hence very logically infer that there must be an infinite spirit as the source and parent principle of the boundless spiritual life of the universe."

The story of the crayon drawing of which the foregoing is a diminished reproduction, plainly and simply told, is as follows: We have from time to time in these columns, made allusion to a class of sensitives, who are endowed with faculties so keenly alive to, and in such harmony with the spiritual, as to form intermediates of intelligible communication between the seen and the unseen worlds; that through their instrumentality, the denizens of the two worlds may not only intercommunicate, but interact by the employment of forces no less satisfactory of result, because incomprehensible of method.

Mrs. Harriet E. Beach is a middle-aged lady, the wife of a prominent scientific gentleman of New York City, very well-known in literary and artistic circles. For a number of years she has devoted herself to the investigation of occult matters, being largely assisted in this by her own mediumistic powers. There is indeed no phase of occult phenomena, with which she is unacquainted. Her private apartments at her city residence constitute a museum of curiosities in this line, so mysterious to most minds. For three years the intelligence represented by the beforementioned drawing has manifested himself to Mrs. Beach in various ways, by means of different medial agencies. He gives his name as Amarona, and represents himself as having lived in the seventh century, A. D., in Egypt, and as having been an alchamist, astrologer, and magician of that remote period, when it is known that persons of his profession were among the most learned in the State, to whom was accorded great distinction. Latterly, at the residence of one of our best known sensitives, this distinguished personage (for we must needs speak of him as such) has presented himself to Mrs. Beach, in tangible form, on no less than six different occasions, and conversed with her with the familiarity of an old acquaintance, as he in truth was. It so happened that on at least one of these occasions there were present Doctor and Mrs. Henry Rogers, two well-known sensitives, of whom it is unnecessary in this place to give a more extended account than to say that, through their medial istrumentality, some of the most marvelous art and psychographical phenomena of modern times have been produced, the *modus operandi* whereof will sufficiently appear in the course of our narrative. The presence was robed in flowing white, bordered with gold, a glittering golden-hued vestment embellished with a double row of hieroglyphics on either side, and a white turban in harmony with the rest. He signified that with the aid of Doctor and Mrs. Rogers he would be able to give Mrs. Beach his picture, after the manner of other monochromatic portraits taken in their presence, a proposition of which the recipient was only too glad to avail herself.

The process ran through a period of ten days, and involved an hour's daily "sitting" by Doctor and Mrs. Rogers, and four "sittings" of the the same period with Mrs. Beach, with no visible results, although it is understood that these preliminary sittings are not alone to harmonize conditions, for it is given out that, during their continuance, the invisible artists are actively employed in producing the picture by methods only known to themselves, and that the final "sitting" is for its *transferrence* upon the material surface provided for it. During these preliminaries, Doctor and Mrs. Rogers were quartered at the Hotel Lafayette, on the south west corner of Broadway and Forty-second Street, New York City. They were to leave for Boston, their present residence, early on the morning of February 1st., and the evening of January 31st. was appointed for the final achievement. The arrangements for it were very simple; an ordinary prepared sheet such as is used for life-size crayon portraits, fastened to a stretcher, was placed upon an easel, which occupied a middle space between the doorway and the rear wall of a small room adjourning a more ample sitting-room, which together constituted Doctor and Mrs. Rogers' hotel apartments, and in a receptacle attached to the easel was placed some finely powdered crayon.

These were all the appliances in the room which could be made available in producing the likeness. The only persons present were Doctor and Mrs. Rogers and Mrs. Beach. They ranged themselves about the doorway leading to the smaller room, which was now curtained off by loosely falling drapery. Almost immediately Dr. Rogers entered into the trance state, being subjected for the time being to the control of an ancient spirit who gives his name as Esmond, who, after a few words explanatory of the divine purpose in permitting the contemplated manifestation, offered a solemn invocation wherein he implored the aid of the Great Spirit in presenting to the children of earth another link in the chain of evidence which establishes the inestimable truth that man is, indeed, a spiritual being, endowed with spiritual perceptions, which have only to be cultivated and redeemed from his grosser elements, to enable him to obtain a knowledge of the ever increasing excellences which lie within his moral and intellectual grasp not only in this life, but in the life to come.

At the conclusion of the invocation, Doctor Rogers was moved to take his seat just within the doorway on the opposite side of the curtain, which remained sufficiently parted to make his presence visible to the two ladies who maintained their seats as first ordered, and who kept up a running conversation with the Doctor's more familiar control now using his organism, whicn was continued for some minutes after the picture as now imperfectly laid before our readers, was completed. We speak of what we know to be true, for aside from the fact that on a former occasion we were permitted to form one of the "sitters" during a similar manifestion, we were on this January evening received at the Doctor's apartments at the very moment when the likeness was ready to be shown, and before the Doctor had been released from his enforced subserviency to its accomplishment, and we are able to state that each of the three persons most nearly concerned in it at once recognized the likeness of that of the spirit "Amarona," to whose presence in visible form we have made allusion. The drapery, too, is substantially the same, but it will be readily understood that however faithful the reproduction of the life-size and strikingly life-like the crayon, it must necessarily lose much in delicacy of touch and execution, in the processes of photographing and photo-engraving which were required for its diminished reproduction here.

That it required the intermingling of spiritual and material elements for its production, is apparent from the conditions demanded by its projectors. That the preliminary sittings were for the purpose of harmonizing these elements and bringing them into more perfect accordance, as between spirits and mortals present, there can be little doubt. As to its being the likeness of one who lived in a past age, we have no well founded conception, but when it is taken into account that prior to this, some ten or twelve crayon portraits have been taken in like manner through the instrumentality of Doctor and Mrs. Rogers, among which are those of persons long since deceased, of whom no likeness was extant, yet the portrait made, as this was unquestionably made, by invisible agencies, was in every such instance pronounced by those knowing to the fact, a most perfect representation of the individual for whom it was taken, in all outward characteristics, and from this it is fair to infer that the likeness of "Amarona" is of the same category.

If any of our readers should feel disposed to find fault with us for venturing to lay before them these 19th century marvels, which have come to be of daily occurrence, we insist that we should be recreant to our duty, should we fail to keep them advised of these living evidences which conduce to a more rational understanding of man, as an entity, his interior self, spiritual environments and future destiny. Nor are we ready to concede that we have any patrons so wedded to old-time superstitions, as to prefer them to a knowledge of these sublime revelations, in such perfect accord with the spirit of all religious and the enlightened sentiment of this wonderfully progressive age, wherein, as never before, the philosophy of life spans the dark chasm we are wont to call death, and reaches into eternity.

## Figures and Tables

**Figure f1:**